# Brain state-dependent robotic reaching movement with a multi-joint arm exoskeleton: combining brain-machine interfacing and robotic rehabilitation

**DOI:** 10.3389/fnhum.2015.00564

**Published:** 2015-10-16

**Authors:** Daniel Brauchle, Mathias Vukelić, Robert Bauer, Alireza Gharabaghi

**Affiliations:** ^1^Division of Functional and Restorative Neurosurgery and Division of Translational Neurosurgery, Department of Neurosurgery, Eberhard Karls University TuebingenTübingen, Germany; ^2^Neuroprosthetics Research Group, Werner Reichardt Centre for Integrative Neuroscience, Eberhard Karls University TuebingenTübingen, Germany

**Keywords:** robotic exoskeleton, brain-computer interface, brain-machine interface, brain-robot interface, upper limb rehabilitation, functional connectivity, stroke

## Abstract

While robot-assisted arm and hand training after stroke allows for intensive task-oriented practice, it has provided only limited additional benefit over dose-matched physiotherapy up to now. These rehabilitation devices are possibly too supportive during the exercises. Neurophysiological signals might be one way of avoiding slacking and providing robotic support only when the brain is particularly responsive to peripheral input. We tested the feasibility of three-dimensional robotic assistance for reaching movements with a multi-joint exoskeleton during motor imagery (MI)-related desynchronization of sensorimotor oscillations in the β-band. We also registered task-related network changes of cortical functional connectivity by electroencephalography via the imaginary part of the coherence function. Healthy subjects and stroke survivors showed similar patterns—but different aptitudes—of controlling the robotic movement. All participants in this pilot study with nine healthy subjects and two stroke patients achieved their maximum performance during the early stages of the task. Robotic control was significantly higher and less variable when proprioceptive feedback was provided in addition to visual feedback, i.e., when the orthosis was actually attached to the subject’s arm during the task. A distributed cortical network of task-related coherent activity in the θ-band showed significant differences between healthy subjects and stroke patients as well as between early and late periods of the task. Brain-robot interfaces (BRIs) may successfully link three-dimensional robotic training to the participants’ efforts and allow for task-oriented practice of activities of daily living with a physiologically controlled multi-joint exoskeleton. Changes of cortical physiology during the task might also help to make subject-specific adjustments of task difficulty and guide adjunct interventions to facilitate motor learning for functional restoration, a proposal that warrants further investigation in a larger cohort of stroke patients.

## Introduction

Despite intensive rehabilitation practice, the restoration of arm and hand function for activities of daily living is still not possible in the majority of stroke patients (Dobkin, 2005). To further increase and standardize the amount of therapy required, robot-assisted training was studied in controlled trials without reaching relevant additional benefits over dose-matched physiotherapy yet (Kwakkel et al., 2008; Lo et al., 2010; Mehrholz et al., 2012; Klamroth-Marganska et al., 2014). The most advanced commercially available training system is presumably an active robotic exoskeleton with seven actuated axes (i.e., degrees of freedom), providing antigravity support for the paretic arm and allowing patients with severe impairment to perform task-oriented practice within a motivating virtual environment (Klamroth-Marganska et al., 2014; Kwakkel and Meskers, 2014). This device provided slightly more functional gain for the participating stroke survivors compared to conventional therapy (Klamroth-Marganska et al., 2014). It has been suggested, that such an improvement might also be due to unspecific influences such as increased enthusiasm for novel interventions on the part of both patients and therapists (Kwakkel and Meskers, 2014). However, this robotic training was less effective at restoring arm strength than conventional therapy (Klamroth-Marganska et al., 2014), probably because it was too supportive when providing “assistance-as-needed” during the exercises (Chase, 2014). This is an inherent limitation of active robotic devices providing support on the basis of system dynamics and/or kinematics (Maciejasz et al., 2014).

The scope for recovery when using advanced assistive rehabilitation technology can be improved by complementary approaches that facilitate neuroplastic changes of the sensorimotor system (Di Pino et al., 2014). Neurophysiological parameters might be a means of avoiding slacking; patients are encouraged and robotic movement feedback is provided only when the brain is most responsive for a peripheral input e.g., mediated via the multi-joint exoskeleton. Many studies used surface electromyography to infer the person’s intention to perform a particular movement and applied it as an input signal for robotic assistance (for an overview, see Maciejasz et al., 2014). However, this physiological parameter might be inadequate as a control signal in the targeted patient group due to paralysis and/or abnormally co-activated muscles (Wright et al., 2014), a condition especially relevant in the severely impaired stroke patients who might benefit most from robotic therapy (Klamroth-Marganska et al., 2014).

More recent approaches applied brain signals to control orthotic training devices within the framework of brain-computer/brain-machine interfaces (BCI/BMI) for stroke rehabilitation by providing patient control over the training devices via motor imagery (MI)-related oscillations of the ipsilesional cortex (Buch et al., 2008, 2012; Ang et al., 2011; Gomez-Rodriguez et al., 2011; Shindo et al., 2011; Ramos-Murguialday et al., 2013). These studies successfully linked the training to the patients’ cortical physiology. However, since all these approaches used either single-joint and/or end-effector-based devices rather than a multi-joint exoskeleton, they did not take the more task-oriented practice of activities of daily living into account, which might be provided within the virtual environment of robot-assisted training set-ups (Klamroth-Marganska et al., 2014). However, a direct comparison to dose-matched robot-assisted therapy revealed only similar clinical benefits for BCI/BMI interventions at best (Ang et al., 2011, 2014).

Although the initial results using BMI were promising (Ang et al., 2011, 2014; Ramos-Murguialday et al., 2013; Pichiorri et al., 2015), clinical improvements for the group of severely affected and chronic patients with no hand use are either still non-existent (Buch et al., 2012) or limited to the arm (Ramos-Murguialday et al., 2013). Particularly with regard to gains in hand and finger function—the main aim of these interventions—no relevant improvements in those patients who were unable to use their hand were observed (Ramos-Murguialday et al., 2013). And so the search for an effective therapy for these patients continues.

Previous BMI interventions served rather as a general priming mechanism for the following physiotherapy training, just as brain stimulation techniques are applied before the subsequent rehabilitation exercises (Ramos-Murguialday et al., 2013; Pichiorri et al., 2015). The open research question is whether such an intervention could be translated into a more task-oriented rehabilitation exercise on the basis of the natural physiology for closing the sensorimotor loop, i.e., by blurring the boundaries between BMI and robotic rehabilitation towards brain-robot interface (BRI) rehabilitation. Bearing this in mind, we tested the feasibility of providing three-dimensional robotic assistance for task-oriented training with the multi-joint exoskeleton during brain-states in which both the participant’s effort to move and the responsiveness of the brain for peripheral input were reflected. This entailed the use of a closed-loop set-up that provided robotic reaching movement following a predefined trajectory during desynchronization of sensorimotor oscillations in the β-band, the prominent natural frequency mediating cortico-muscular communication (van Wijk et al., 2012; Kilavik et al., 2013). In addition, we hypothesized that controlling a robot-assisted reaching movement through regional modulation of β-band oscillations is a cognitively demanding task that leads to additional network changes of cortical functional connectivity. Our aim was to capture these changes via the corrected imaginary part of the coherence function, i.e., a robust connectivity measure ignoring relations at zero phase lag and therefore insensitive to volume conduction properties (Nolte et al., 2004; Ewald et al., 2012).

## Materials and Methods

### Study Design and Participants

We recruited nine right-handed healthy subjects (S1–9, two female, mean age = 26 ± 4 years), and two right-handed stroke survivors (P1–2, both male): P1 (52y) had suffered an ischemic stroke of the right hemisphere 156 months earlier, while P2 (56y) had suffered a hemorrhagic stroke of the right hemisphere 78 months before enrollment. Neither patient had functional control of their left upper extremity (Medical Research Council motor scale <2) and a persistent hemiparesis with 18 points (P1) and 25 points (P2) out of a maximum of 54 points in the modified Fugl-Meyer Assessment Upper Extremity (FMA-UE) score (Ramos-Murguialday et al., 2013), respectively. All subjects gave their written informed consent to participate in a study, which was approved by the ethical review committee of the local medical faculty and involved reaching training with a multi-joint exoskeleton of the right (S1–9) or left arm (P1–2).

Six healthy subjects and two stroke patients performed the first BRI task. Each participant’s arm was attached to the orthotic exoskeleton. Proprioceptive, haptic and visual feedback was administered with respect to β-ERD in the MI phase (proprioceptive condition).

Three healthy subjects performed a second BRI task which was identical to the first one except that their arms were not attached to the exoskeleton. The subjects controlled the robotic arm with sensorimotor β-ERD just as if it were an external prosthesis. They then observed the respective movement (visual condition).

We compared these two task conditions of controlling the multi-joint exoskeleton since our previous work suggests that proprioceptive feedback is superior to visual feedback only in facilitating brain self-regulation of β-ERD (Vukelić and Gharabaghi, 2015a).

**Figure 1:**
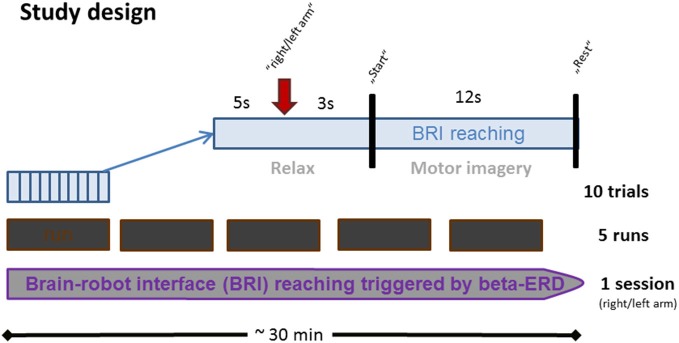
**Time course of BRI session.** Each BRI session lasted for approximately 30 min and consisted of five runs which were interrupted by four short breaks. Every run consisted of 10 trials, each lasting 20 s. In each trial, subjects were instructed to prepare for MI upon an auditory cue (3 s preparation phase), and to imagine the respective reaching movement upon a “start” cue (12 s MI phase). This was followed by a “rest” cue (5 s rest phase).

Each session lasted approximately 30 min and consisted of five runs, each consisting of 10 trials (Figure 1). In every trial, subjects were instructed by an auditory cue to prepare for MI cue (3 s preparation phase), and to imagine the reaching movement following a “start” cue (12 s MI phase), which was followed by a “rest” cue (5 s rest phase).

### Robotic Exoskeleton

**Figure 2:**
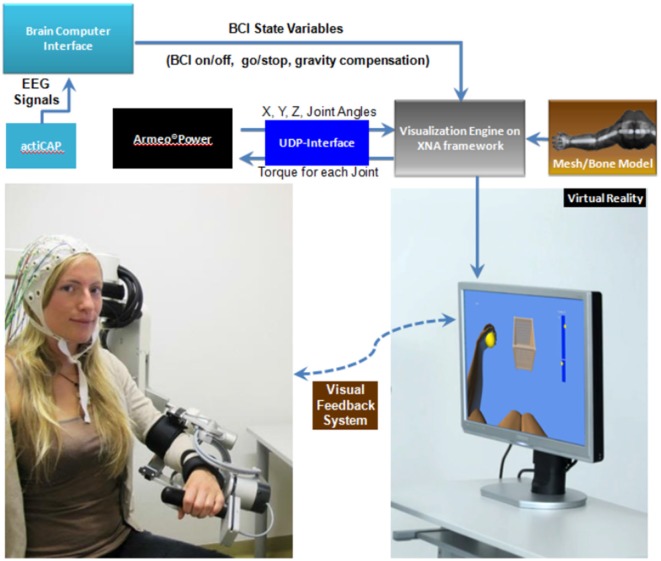
**Schematic overview of the setup.** EEG signals from an ActiCAP system are analyzed by the BCI software (BCI2000) which controls the visualization engine. The robot’s end-effectors position and its joint angles are transmitted to the virtual reality (VR) framework via a UDP interface. This information is integrated with a mesh model of the upper extremity, allowing for 3D virtual movements in real time.

**Figure 3:**
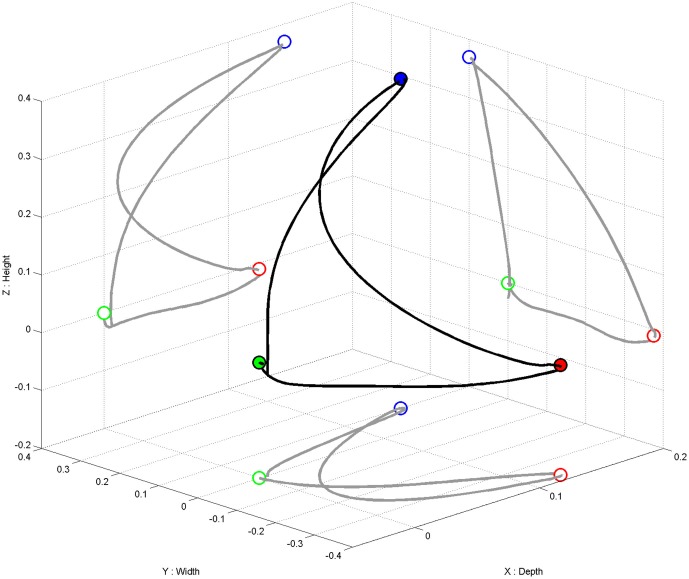
**Movement trajectory in three-dimensional space (in black).** Projection (in gray) of the three-dimensional movement on the respective two-dimensional planes (i.e., axis-surfaces *x/y*, *x/z*, *y/z*). Starting point (i.e., target 3) represented as a green dot, target 1 as a red dot, target 2 as a blue dot.

We used the commercially available version (ArmeoPower, Hocoma, Volketswil, Switzerland) of the rehabilitation robot recently studied in a controlled stroke trial (Klamroth-Marganska et al., 2014). This device is an active robotic exoskeleton for shoulder, elbow and wrist joints with seven actuated axes (i.e., degrees of freedom). It provides antigravity support for the paretic arm and enables patients with severe impairment to perform task-oriented practice within a motivating virtual environment (Figure 2). The safety of operations was ensured by the ArmeoControl 1.23 software which was extended by an in-house developed plugin. Six of the seven motor-actuate joints could be controlled during the task, while the seventh was adjusted only once to account for the participant’s body height and seating position before the onset of training. The most distal sensor was a pressure sensitive grip which assessed the grasping force of the user’s hand (Bishop and Stein, 2013). Using a User Datagram Protocol (UDP)—Interface, a custom-made software plugin interfaced the robot’s actions from external network devices while receiving time-stamped (ID-tagged) messages with information about the current device state from the robot. This information consisted of 17 floating-point numbers composed of message-ID, angular position of each controllable joint, mechanical torque applied by the individual motors, *x*-, *y*-, *z*-location of the end effector and the grip pressure. The packages were transmitted to the network at a rate of 50 times per second. All data was stored in a text file on the measurement PC and then used to provide online visual feedback in a virtual reality (VR) environment based on the Microsoft XNA Gamestudio 3.0 and written in C#. The VR environment continually received time-stamped packages containing all joint angles of the exoskeleton’s current joint configuration. The upper extremity shown in the VR was concurrently updated according to the robot’s position. A robotic movement of the subject’s arm in three-dimensional space (Figure 3, right side) could therefore be traced in VR (Figure 3, left side). The task in this study was to reach three targets, all of which required consecutive reaching movements of the arm, i.e., performing a reaching movement to the side and forward (target 1), followed by a reaching movement upward (target 2) and a retrieving movement backward (target 3). The targets were represented by spheres which changed color once they were reached. The robot was programmed to move with the participant’s arm on a pre-defined trajectory—once per trial—from the starting position (target 3) to target 1, from there to target 2, and from target 2 to target 3, i.e., back to the starting position. This robotic movement was brain-state dependent, i.e., it was performed only during β-band desynchronization in the MI phase. After the imagery phase, the robot always returned to the starting position (target 3) if it had not reached its target by the end of the allocated 12 s.

### Brain-Robot Interface and Data Acquisition

Scalp EEG potentials were taken (BrainAmp, Brainproducts GmbH, Germany) from 64 positions in accordance with the international 10–20 system with Ag/AgCl electrodes (ActiCAP, Brainproducts, GmbH, Germany). All impedances were kept below 20 kΩ. Following digitization at 1 kHz rate and high-pass filtering with a time constant of 10 s the EEG signals were transferred to the BCI2000 software (Schalk et al., 2004) for online analysis, triggering the robot and offline storage. Participants controlled the brain-robot interface (BRI) by volitional control of their sensorimotor oscillations in the β-band during the MI phase (Gharabaghi et al., 2014a; Vukelić et al., 2014). Following calibration, the regional oscillatory power of the frequency band from 16–22 Hz over the EEG electrodes contralateral to the moving arm (i.e., FC5, C5, CP5 or FC4, C4, CP4) was estimated online, and the event-related desynchronization in the β-band (β-ERD) was classified by a linear classification algorithm. This entailed estimating every 40 ms frequency power over the preceding 500 ms using an autoregressive model based on the Burg Algorithm with a model order of 32 (McFarland and Wolpaw, 2008). We used nine features for our classification scheme. These consisted of 2-Hz frequency bins of β-ERD during the MI phase relative to the average power of the rest phases of the last 15 s and three EEG electrodes overlying sensorimotor areas contralateral to the movement imagination (Gharabaghi et al., 2014a; Vukelić et al., 2014). On the basis of the classification result, the robot moved the arm contingently following a predefined trajectory at a predetermined speed or ceased its movement. In the rest phase, the robot returned to the starting position.

The cortical physiology (β-modulation range and functional cortical networks) was analyzed for the patients (*n* = 2) and for the healthy subjects with proprioceptive feedback (*n* = 6). One of these healthy subjects exhibited channels with large artifacts, preventing the inclusion of this data in the physiological analysis. Thus, the cortical physiology was studied for two patients and five healthy subjects in the proprioceptive condition.

### Data Preprocessing

Artifacted EEG electrodes, as determined by visual inspection, were excluded from further analysis. We focused on two temporal windows for the analysis of the functional connectivity networks: rest (baseline) epoch (from −6 s to −3 s and MI epoch. The MI epoch was further subdivided into two 3 s epochs (from 1 s to 4 s referred to as first MI epoch, and from 9 s to 12 s referred to as last MI epoch). Epochs containing a maximum deviation above 100 μV in any of the EEG channels were discarded (Sanei and Chambers, 2008). The functional cortical networks were calculated by detrending the EEG signals, zero-padding and band-pass filtering between 1 Hz to 42 Hz.

Event-related spectral perturbation (ERSP) was calculated by band-pass filtering the signals between 14 and 24 Hz. The filtering procedures were performed with a first order zero-phase lag FIR filter. We also carried out an independent component analysis (ICA) using the logistic infomax ICA algorithm as implemented in the EEGLAB toolbox (Delorme and Makeig, 2004), identified components with the remaining artifacts by visual inspection and removed them from the signal.

### Estimation of Robotic Movement

The position of the exoskeleton end point in space was measured in three dimensions (*x*, *y*, *z*) with a 25 Hz sampling rate. The Euclidian vector norm of the change in three-dimensional position was then calculated between subsequent samples for movements larger than 0.01 cm, resulting in a movement probability for each sample. The average probability of robotic movement was calculated as the average of movement occurrence in the MI phase (0–12 s). Thus, movement probability was computed as the relative ratio of the number of trials with movement greater than 0.01 cm at each time point, i.e., a robotic movement value of 40% at a particular time point indicates that for 40% of the trials, there was a movement larger than 0.01 cm at that particular time point. To increase signal-to-noise ratio, the time course was smoothened with a low-pass filter for frequencies below 5 Hz.

### Estimation of β-Modulation

The individual β-modulation range for each subject and patient was calculated as reported recently (Vukelić et al., 2014; Vukelić and Gharabaghi, 2015a). The same frequency band and EEG electrodes that had been used for self-regulation and neurofeedback were analyzed. To estimate the β-modulation range, we carried out an off-line calculation of the ERSP between 16 and 22 Hz with a frequency resolution of 0.24 Hz for each electrode, as implemented in the EEGLAB toolbox (Delorme and Makeig, 2004). In a first step, the ERSP for each channel was calculated trial-wise and normalized with respect to the rest baseline. In the second step, we averaged the ERSP for every trial across channels. In a third step, we averaged the ERSP across all trials. Finally, we selected the frequency bin with the largest range between the *maximum* in the rest epoch (describing the maximum synchronization potential) and the *minimum* in the MI epoch (describing the maximum de-synchronization potential). This measure was used as indicator for the individual’s ability to modulate sensorimotor β-activity during the BRI task.

### Estimation of Functional Cortical Networks

To calculate functional connectivity, we used the corrected version of the imaginary part of coherence (ciCOH; Ewald et al., 2012). This measure disregards relations at zero phase lag and is therefore insensitive to volume conduction properties, thus indicating the relative coupling of phases, i.e., the time-lag between two brain processes. This version also has additional features that compensate for preference for remote interactions by increasing SNR. This enables us to observe interactions that are otherwise hidden in the noise when studying connectivity between sensors. Since the ciCOH is based on an estimation of the complex coherency function, each valid epoch was subdivided into segments of 1 s length with 50% overlap. This corresponds to a frequency resolution of δf = 1 Hz (Nolte et al., 2004). Each segment was then multiplied with a Hanning window. A Fourier transformation of the data provided an estimation of the cross-spectra between two time-series (Nolte et al., 2004). The complex coherency function was defined as the normalized cross-spectrum for channels *i* and *j*, respectively:

(1) $$C_{ij}(f)=\frac{{\text{S}}_{ij}(\text{f})}{\sqrt{{\text{S}}_{ii}(\text{f}){\text{S}}_{jj}(\text{f})}}$$

where *S_ij_*(·) represents the cross-spectrum between channels *i* and *j*, and *S_ii_*(·), *S_jj_*(·) represents the auto-spectra for channels *i* and *j*, respectively. We systematically evaluated the functional connectivity networks between the contralateral M1 motor network and the entire brain (all other EEG channels) by defining a seed electrode in contralateral or ipsilesional primary motor cortex (C5 for the healthy subjects and C4 electrode for the stroke patients). The ciCOH function was thus calculated from the complex coherency function (Ewald et al., 2012)

(2) $$ciCOH_{\text{seedj}}(f)=\frac{\text{Im}\left({\text{COH}}_{\text{seedj}}(\text{f})\right)}{\sqrt{\left(1-\text{Re}{\left({\text{COH}}_{\text{seedj}}\right)}^{2}\right)}}$$

where *Seed* denotes the seed electrode, *f* indicates frequency bins and *lm*(·) and *Re*(·) denote the imaginary and real parts, respectively. The ciCOH was further Fisher z-transformed to fit a Gaussian distribution (Rosenberg et al., 1989; Nolte et al., 2004). We evaluated the functional connectivity within predefined frequency bands of interest (FOI). The frequency bands were thus defined as follows: θ-band (3–7 Hz), α-band (8–14 Hz), lower β-band (15–25 Hz) and upper β-band (26–40 Hz). In a next step, the functional connectivity measure was obtained by averaging the absolute value of ciCOH across frequencies within each predefined FOI. We statistically evaluated the functional connectivity as the difference between the two MI epochs (3 s) and baseline (rest) epoch. All data analysis was performed offline with custom written scripts in MATLAB^®^.

The cortical physiology (β-modulation range and functional cortical networks) was analyzed for the two BRI tasks with proprioceptive feedback.

### Statistics

For statistical analysis of the time course of robotic movement during the imagery phase, non-parametric tests were employed to account for the low sample size and possibility of non-normal distributions. Differences between the visual and proprioceptive feedback groups were tested with a Kruskal-Wallis test. Each individual patient was contrasted with the proprioceptive feedback group based on a sign-test. The false discovery rate for all statistical comparisons was limited to 5%.

The statistical analysis of the functional connectivity networks (MI vs. baseline epochs) involved a non-parametric cluster-based permutation analysis to account for the low number of samples (i.e., subjects) and the subsequent possibility of non-normality (Benjamini and Hochberg, 1995; Nichols and Holmes, 2002; Maris, 2012). This approach also enabled us to incorporate neurophysiologically motivated constraints to the test statistic (i.e., spatially clustering neighboring electrodes) while controlling for the family-wise error rate and correcting for the multiple comparison problem (Nichols and Holmes, 2002; Maris and Oostenveld, 2007; Maris et al., 2007; Maris, 2012). This involved a cluster-based non-parametric randomization procedure as implemented in FieldTrip (Maris et al., 2007; Oostenveld et al., 2010).

Multiple *dependent* sample *t*-statistics were carried out on the healthy subjects to establish the topography of functional connectivity differences. We applied critical sensor *t*-values for spatial clustering of neighboring electrodes with* a priori* threshold of *p* < 0.05. The cluster level statistics were defined as the sum of *t*-values within each cluster. Multiple comparisons were corrected by calculating the 95th percentile (two-tailed) of the maximum values of summed *t*-values estimated from an empirical reference distribution. Any *T*-values exceeding this threshold were considered as significant at *p* < 0.05 (corrected). A permutation test (randomly permuting the data across MI and baseline epochs 1000 times) was used to calculate the reference distribution of maximum values. This enabled us to evaluate the statistics of the actual data for each FOI individually.

A multiple *independent* sample *t*-test was used for the patients. The cluster level statistics were defined as the sum of *t*-values within every cluster by spatially clustering neighboring electrodes on the basis of an* a priori* threshold of *p* < 0.05. The correction for multiple comparison was calculated for the 95th percentile (two-tailed) of the maximum values of the summed *t*-values while considering *t*-values at *p* < 0.05 (corrected) as significant. The null distribution was achieved by randomly permuting the data between the MI and baseline epochs 1000 times.

## Results

**Figure 4:**
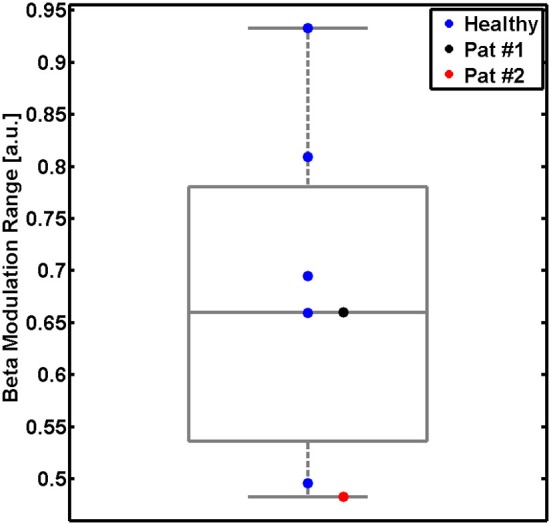
**β-modulation range during the proprioceptive feedback condition.** Modulation range for five healthy subjects (single blue dots and group results as boxplot) and for two stroke patients (black and red dots). This measure reflects the individual ability to modulate sensorimotor β-oscillations captured with the feedback electrodes. The β-modulation range is defined in the individual frequency bin of the event-related spectral perturbation (ERSP) with the largest difference between the motor imagery (MI) epoch (the minimum) and in the rest epoch (the maximum).

**Figure 5:**
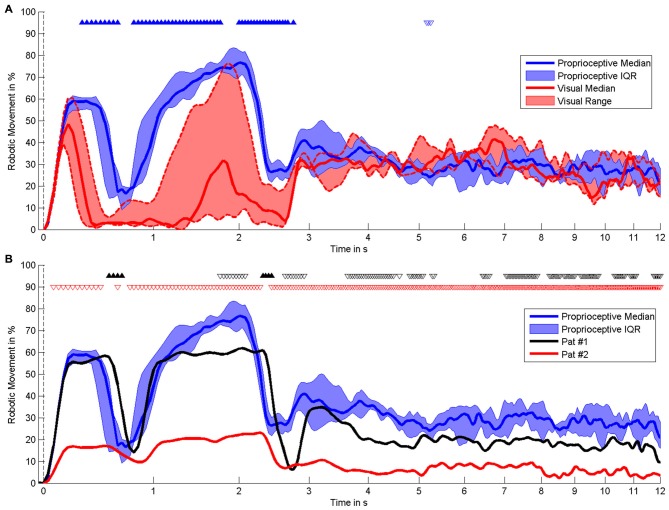
**Time course of robotic movement.** It shows the occurrence of robotic movement with time on the x-axis (logarithmic scale) and probability of movement on the y-axis. The MI phase begins at 0 s. Smoothing was performed by low-pass filtering below 0–5 Hz. **(A)** Comparison of different feedback modalities in healthy subjects, i.e., proprioceptive feedback (*n* = 6) vs. visual feedback (*n* = 3). **(B)** Comparison of individual stroke patients with proprioceptive feedback with healthy subjects with proprioceptive feedback (*n* = 6). The results of the statistical comparison are indicated by triangles, i.e., filled triangles pointing upwards and empty triangles pointing downwards indicate significantly higher and lower movement probability, respectively. Black and red triangles are applied to compare patients #1 and #2 with healthy subjects.

The present pilot study revealed the feasibility of controlling three-dimensional robotic assistance for task-oriented training with a multi-joint exoskeleton by the modulation of movement-related natural brain-states. Both healthy subjects and stroke survivors were able to modulate sufficient sensorimotor ERSP in the β-band for this purpose, as indicated by the β–modulation range (Figure 4). Surprisingly, all participants had very similar patterns of robot control regardless of the clinical condition (healthy/patient) or the feedback modality (proprioceptive/visual): the first peak of robot movement occurred within the first second, another, longer peak was observed between the first and third second and a third peak occurred about three seconds after the onset of the MI phase (Figure 5). During these first three seconds of the feedback period (see Figure 5A), healthy subjects showed a significantly higher average performance of robotic control in the proprioceptive as compared to the visual *condition* χ^2^(7, 1) = 5.4, *p* = 0.02). The patients showed significantly lower average performances than their healthy counterparts with proprioceptive feedback for the full feedback duration (*s*_(6)_ = −6, *p* = 0.0313).

During later periods of the MI task, i.e., between four and twelve seconds after the start, the performance dropped again and remained relatively unchanged for all participants. While the stroke patients also performed significantly less well than healthy subjects in these later task periods, i.e., similar to the early task periods (see Figure 5B), the healthy subjects no longer showed any systematic differences between the proprioceptive and the visual condition (see Figure 5A).

**Figure 6:**
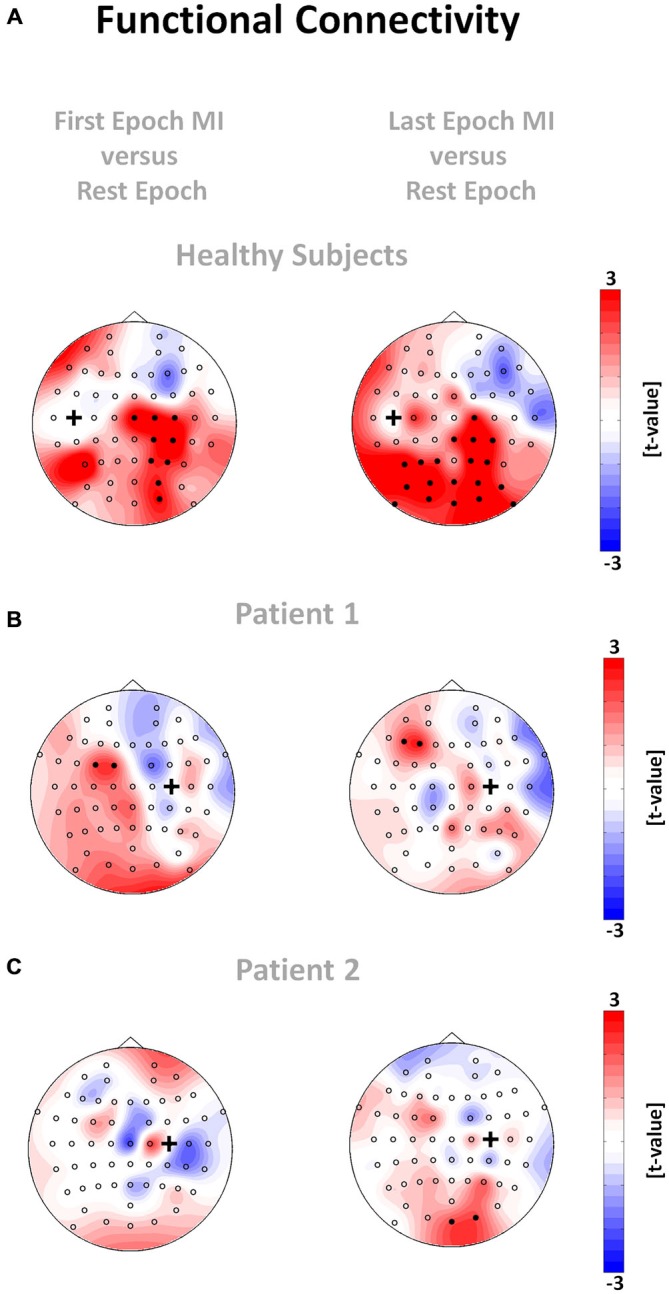
**Functional connectivity networks for healthy subjects (A) and patient 1 (B) and 2 (C).** The plots show the t-value topographies of functional connectivity (absolute value of ciCOH) as a contrast between the two MI epochs and the rest epoch for the θ-band. The black cross indicates the seed electrode position. Electrode clusters, showing significant differences in the non-parametric randomization test, are indicated by filled black circles. Colors indicate functional connectivity increases (red) and decreases (blue) within the MI epoch relative to the rest epoch.

The regional modulation of β-band oscillations led to significant task-related network changes of cortical functional connectivity, with relevant differences between healthy subjects and stroke patients and between early and late periods of the task: In healthy subjects, functional connectivity in the θ-band during the MI epoch was higher than in the rest epoch. These different functional connectivities during the rest and task epochs revealed a specific topographic pattern visualized on a *t*-value scale (non-parametric randomization test) across all subjects for the motor cortical network. More specifically, we observed that the seed electrode over the left motor cortex, which was volitionally modulated in the β-band during the feedback task, showed stronger functional connectivity with electrodes over right sensori-motor and parieto-occipital regions in the θ-band in healthy subjects (Figure 6A, left plot). At later stages of the task, this functional connectivity in the θ-band even increased bi-laterally to include extended parieto-occipital regions (Figure 6A, right plot). None of the patients showed a comparable pattern of distributed coupling. Throughout the whole task period (Figure 6B) P1 presented a circumscribed increase of θ-band connectivity between the seed electrode over the ipsilesional motor cortex, which was volitionally modulated in the β-band, and electrodes at contralesional fronto-premotor regions. P2 showed significant θ-band connectivity between the seed electrode of the ipsilesional motor cortex and electrodes over occipital regions only (Figure 6C).

## Discussion

This study has illustrated that brain-robot interfaces (BRIs) may successfully link three-dimensional robotic training to the participant’s effort and allow for task-oriented practice of activities of daily living with a physiologically controlled multi-joint exoskeleton. Both healthy subjects and stroke survivors showed similar patterns of robot control, with the maximum performance in the early period of the task and a significant drop during later periods. As the movement trajectory was defined by three targets, it would be plausible to relate the findings to discrete movement planning connected to reaching the intermediate targets. The significant performance change, however, occurred after ~3 s, while the average time period to reach target 1 was ~5 s. This indicates that the performance drop was not related to task adjustments during the transition from target 1 to target 2, i.e., not linked to discrete movements. The occurrence of three movement peaks within the early task period conflicts with the interpretation of this finding as a pure sensorimotor transitory phase from rest to movement as well. Moreover, this pattern was independent of the clinical status and the feedback modality. These findings indicate that the observed pattern might be typical for this kind of tasks, i.e., complex arm movement, and suggest that the task length of future training protocols needs to be adjusted to the time period in which participants are able to sustain brain states of β-ERD.

In this context it should be considered that previous studies showed that proprioceptive robotic feedback activated a distributed cortical network (Vukelić et al., 2014) lasting beyond the intervention period (Vukelić and Gharabaghi, 2015b), bridged the gap between the abilities and cortical networks of MI and motor execution (Bauer et al., 2014), and facilitated—in comparison to visual feedback—sensorimotor β-ERD (Vukelić and Gharabaghi, 2015a) thereby improving brain state classification between rest and movement imagery (Gomez-Rodriguez et al., 2011). When interpreting the present findings along these lines, proprioceptive feedback seems to facilitate MI-related β-ERD only during the first few seconds of a feedback task. Sustaining β-ERD is thus challenging and may even be characterized by a significant association with the experience of frustration for the participants (Fels et al., 2015). Therefore, the subject’s cognitive resources for coping with the mental load that occurs during such a neurofeedback task needs to be considered (Bauer and Gharabaghi, 2015a; Naros and Gharabaghi, 2015). Mathematical modelling on the basis of Bayesian simulations indicates that this might be achieved when the task difficulty is adapted in the course of the training (Bauer and Gharabaghi, 2015b). Such an adaptation strategy would facilitate the patient’s reinforcement learning by balancing challenge and motivation (Naros and Gharabaghi, 2015).

The limited time period, in which participants were able to sustain brain states of β-ERD in this study, might also be related to the fact that all participants performed only one training session. Future studies will have to clarify whether performing this task for more that one session might increase the length of MI related β-ERD. Moreover, it needs to be researched in future patient studies how movement intention—instead of MI—would influence physiologically controlled robotic training.

However, in the early stages of the task, robotic control was significantly higher and less variable in the proprioceptive than in the visual condition. This observation might help to resolve the ambiguity of previous BMI studies in healthy subjects. Gomez-Rodriguez et al. (2011) showed that proprioceptive feedback facilitated decoding of MI more efficiently than visual feedback alone. By contrast, Ramos-Murguialday et al. (2012) reported no group level improvements in performance when MI was coupled with contingent positive proprioceptive feedback compared to MI without feedback. This ambiguity might be related to the way in which MI was decoded in these studies. While Ramos-Murguialday et al. (2012) used sensorimotor α-band (8–12 Hz) activity to differentiate between MI and rest, Gomez-Rodriguez et al. (2011) examined both α-band and β-band activity and ascertained that the best discriminative power for the proprioceptive condition in healthy subjects was in the β-band. However, Gomez-Rodriguez et al. (2011) applied a support vector machine to analyze 20 frequency bins (2–42 Hz) for each of 35 bilaterally distributed recording channels, i.e., a 700-dimensional feature space, for the online task, which thus lacked spatial and spectral specificity. By focusing on the β-band (16–22 Hz) from three channels of the sensorimotor cortex, we restricted the feature space to nine features, thereby proving their specificity for the performance gain by proprioceptive feedback. These results complement observations that providing visual feedback of MI related β-oscillations increased the laterality at the targeted contralateral sensorimotor cortex (Boe et al., 2014) and the movement-related desynchronization in this frequency band (Bai et al., 2014). Moreover, the present findings are line with the recent observation that proprioceptive feedback is superior to visual input for increasing self-control of regional β-band modulation (Vukelić and Gharabaghi, 2015a) and that such a BRI might bridge the gap between the abilities and cortical networks of MI and motor execution (Bauer et al., 2014).

As anticipated, stroke patients performed considerably less well than healthy subjects in controlling the robotic movement with β-ERD. This finding might be related to age differences between these groups. However, the very similar pattern of robot control between healthy subjects and patients, i.e., with the maximum performance in the early period of the task and a significant drop during later periods, suggests that age did at least not influence the general time course of the human-robot interaction. A closer look at the β-modulation range (see Figure 4) also indicates that each of the patients was matched by one healthy participant with the very same (limited) capability of brain self-regulation. Furthermore, an earlier study of our group (unpublished data) demonstrated that the skill of self-regulating regional sensorimotor β-oscillations in a BRI environment might be independent of whether MI of the dominant or non-dominant hand is performed, thus suggesting that the observations of this study are probably not related to which hand the healthy subjects and patients used to carry out the task. However, future studies will have to consider these aspects before drawing definite conclusions.

In the physiological domain, earlier studies had already shown a higher discriminative power of α-band activity in this patient group (Gomez-Rodriguez et al., 2011). Therefore, previous studies—based on a long tradition of BCI/BMI research—chose those frequency bands and algorithms for controlling these devices that differentiated best between “MI” and “rest”, e.g., the mu/alpha-band, modified common spatial filter algorithms and/or machine learning techniques, to optimize the selection of temporo-spatial discriminative oscillatory characteristics (Buch et al., 2008, 2012; Ang et al., 2011, 2014; Gomez-Rodriguez et al., 2011; Ramos-Murguialday et al., 2013; Pichiorri et al., 2015). Although even larger groups of stroke patients have participated in training with this “classical” BCI/BMI approach, a more restricted feature space based on neurophysiological considerations—namely perturbations in the β-band over selected sensorimotor electrode contacts—might be preferable as a reinforced therapeutic target for restorative purposes (Gharabaghi et al., 2014a,b,c; Naros and Gharabaghi, 2015). Such an approach, albeit less optimal for classification purposes to differentiate movement-related brain states in stroke patients (Gomez-Rodriguez et al., 2011; Rossiter et al., 2014), might approximate the physiological situation of closing the sensorimotor loop further (van Wijk et al., 2012; Kilavik et al., 2013). However, only future intervention studies with more stroke patients can clarify whether any functional benefits are to be gained by training this specific physiological marker. Further studies with a larger group of patients are also needed to determine the extent to which the *electroencephalography*-based approach presented here will be of benefit to complex real-life activities if control is limited to the movement of the robotic arm, but not to its trajectory. Applying an alternative approach for stroke rehabilitation closer to the neural signal source with epidural *electrocorticography* (ECoG) might overcome this limitation (Gharabaghi et al., 2014a,b). We recently showed that up to seven hand movement intentions could be decoded with sufficient accuracy when this ECoG approach is applied in severely affected chronic stroke patients with no residual hand function (Spüler et al., 2014).

To gain a better understanding of the neurophysiological correlates of the BRI technique applied here, we also studied the distributed functional network architecture of the cortical motor system. We discovered significant networks in the θ-band during the task as well as differences in these θ-networks between healthy subjects and stroke patients and between early and late periods of the BRI task.

We successfully demonstrated that interhemispheric communication in healthy subjects was mediated in the θ-frequency band, revealing increased functional connectivity of the seed electrode over the primary motor cortex of the left hemisphere with the homologous cortex of the right hemisphere, i.e., with electrodes over right sensori-motor and parieto-occipital areas during the early period of the task. In later task periods, when the performance of robot control dropped significantly, left hemispheric parieto-occipital areas also revealed a coupling. These findings are in line with our recent observation that participants with a lower ability to maintain prolonged states of β-ERD during MI and feedback with a hand robot recruited a learning related “scaffolding” θ-band network between the motor cortex and the parieto-occipital cortex to a higher degree (Vukelić and Gharabaghi, 2015a). Moreover, these results are in accordance with the known physiology of movement preparation and execution: the recruitment of homologs sensorimotor regions is essential for motor control and motor skill learning (Beaulé et al., 2012; Takeuchi et al., 2012). The link to occipital regions very probably mediates the visuomotor integration during the task (Suminski et al., 2010; Wu et al., 2014). Finally, the recruitment of left parietal regions is essential for multisensory integration of visual somatosensory and proprioceptive information in the context of planning and controlling voluntary movements (Lloyd et al., 2006; Desmurget and Sirigu, 2009). Thus, controlling the BRI with sensorimotor oscillations resulted in the recruitment of distributed and functionally coupled motor related areas that are typically activated during natural movements.

This pattern was not detected in either of the stroke patients, indicating the known cortical reorganization of intra- and interhemispheric communication related to impaired motor and cognitive behavior (Rehme et al., 2010, 2011; Dubovik et al., 2012). However, one of the patients (P1) showed a circumscribed increase of θ-band connectivity between the seed electrode of the ipsilesional motor cortex, which controlled the BRI, and the electrode at contralesional fronto-premotor regions throughout the task. P1 had a significantly higher ratio of robotic movement (*t*_(183)_ = 5.43, *p* < 0.001, *δ* = 0.17, CI95*%* = 0.11–0.23) than P2, particularly during the first four seconds of the task (see Figure 5B), indicating a significantly better control of the BRI. This observation matches previous findings relating the interaction between primary motor and fronto-premotor regions, such as premotor and supplementary motor regions (SMA), to skill acquisition (Hikosaka et al., 2002; Vahdat et al., 2011) and in particular SMA to BCI control (Halder et al., 2011). Moreover, studies with stroke patients have revealed a relationship between the recruitment of contralesional premotor/supplementary motor proportional to the clinical impairment (Bestmann et al., 2010; Ward, 2011).

The results presented here very probably reflect a cross-frequency interaction between regional β-band and distributed θ-band activity which has also been observed during cognitively demanding tasks (Palva et al., 2005). For such interactions, there would appear to be a hierarchy in which the lower frequencies modulate the higher ones (Jensen and Colgin, 2007; Canolty and Knight, 2010). This may provide us with the opportunity to improve the BRI performance by modulating the θ-band network. Feurra et al. (2013) showed that transcranial alternating current stimulation (tACS) in the θ-range enhances endogenously generated cortical oscillations during movement imagination, thereby leading to increased cortico-spinal excitability. Along these lines, θ-tACS might be used in future studies to modulate motor learning and working memory related networks (Polanía et al., 2012) before or in parallel with BRI rehabilitation. Moreover, changes in cortical physiology during the task might facilitate subject-specific adjustments of task difficulty and guide adjunct interventions to facilitate functional restoration.

## Conflict of Interest Statement

The authors declare that the research was conducted in the absence of any commercial or financial relationships that could be construed as a potential conflict of interest.
